# Measurement Modulus of Elasticity Related to the Atomic Density of Planes in Unit Cell of Crystal Lattices

**DOI:** 10.3390/ma13194380

**Published:** 2020-10-01

**Authors:** Marzieh Rabiei, Arvydas Palevicius, Amir Dashti, Sohrab Nasiri, Ahmad Monshi, Andrius Vilkauskas, Giedrius Janusas

**Affiliations:** 1 Faculty of Mechanical Engineering and Design, Kaunas University of Technology, LT-51424 Kaunas, Lithuania; andrius.vilkauskas@ktu.lt; 2 Department of Materials Science and Engineering, Sharif University of Technology, Tehran 11365-9466, Iran; a.dashty@merc.ac.ir; 3 Department of Materials Engineering, Isfahan University of Technology, Isfahan 84154, Iran; a-monshi@cc.iut.ac.ir

**Keywords:** Young’s modulus, X-ray diffraction, planar density, crystalline materials, elastic compliances, modified W–H

## Abstract

Young’s modulus (E) is one of the most important parameters in the mechanical properties of solid materials. Young’s modulus is proportional to the stress and strain values. There are several experimental and theoretical methods for gaining Young’s modulus values, such as stress–strain curves in compression and tensile tests, electromagnetic-acoustic resonance, ultrasonic pulse echo and density functional theory (DFT) in different basis sets. Apparently, preparing specimens for measuring Young’s modulus through the experimental methods is not convenient and it is time-consuming. In addition, for calculating Young’s modulus values by software, presumptions of data and structures are needed. Therefore, this new method for gaining the Young’s modulus values of crystalline materials is presented. Herein, the new method for calculating Young’s modulus of crystalline materials is extracted by X-ray diffraction. In this study, Young’s modulus values were gained through the arbitrary planes such as random (hkl) in the research. In this study, calculation of Young’s modulus through the relationship between elastic compliances, geometry of the crystal lattice and the planar density of each plane is obtained by X-ray diffraction. Sodium chloride (NaCl) with crystal lattices of FCC was selected as the example. The X-ray diffraction, elastic stiffness constant and elastic compliances values have been chosen by the X’Pert software, literature and experimental measurements, respectively. The elastic stiffness constant and Young’s modulus of NaCl were measured by the ultrasonic technique and, finally, the results were in good agreement with the new method of this study. The aim of the modified Williamson–Hall (W–H) method in the uniform stress deformation model (USDM) utilized in this paper is to provide a new approach of using the W–H equation, so that a least squares technique can be applied to minimize the sources of errors.

## 1. Introduction

Young’s modulus is an investigation of the stiffness of elastic materials, so it can be defined as the ratio of stress to strain [1]. The elastic constants are specific to the reaction of the lattice crystal against forces, as determined by the bulk modulus, shear modulus, Young’s modulus and Poisson’s ratio [2]. Elastic constants play a role for determining the strength of the materials [2]. Elastic constant values have a correlation with planar density values due to the bonding characteristic between adjacent atomic planes and the anisotropic character of the bonding and structural stability [3,4]. Elastic constants are created in the relationship between stress and strain and they are dependent on the configuration of crystal lattice, therefore elastic constants are derived from planes in the crystal lattice [5,6,7,8]. Furthermore, there are several studies on the correspondence of elastic constants and planes/directions such as Ref [9,10,11]. Crystallographic planes that are equivalent have similar atomic planar densities. Planar density is the fraction of the total crystallographic plane area that it is occupied by atoms [9]. The planar density is a significant parameter of a crystal structure, and it is specified as the number of atoms per unit area on a plane [12]. X-ray diffraction (XRD) is a conventional procedure to analyze materials. X-ray diffraction can determine the crystalline size, stress, strain and density energy of materials [13]. X-ray diffraction is the only technique that allows the determination of both the mechanical and microstructural states of each diffracted plane. Diffracted planes are utilized as a strain gauge to measure Young’s modulus in one or several planes/directions of the diffraction vector [14]. Nowadays, X-ray diffraction is a conventional technique for the study of crystal structures and atomic spacing. X-ray diffraction is based on constructive interference of monochromatic X-rays and a crystalline sample [15]. In 1969, Hanabusa et al. presented the new method for measuring the elastic constant of cementite phase in steel, but the restriction of this method was related to utilizing the high-angle region only and it was not capable of detecting in low-angle regions [16]. Differently, the Williamson–Hall (W–H) method well corresponded to calculating and estimating strain. The mechanical results extracted by the W–H method were gaining lattice strain (ε), lattice stress (σ) and lattice strain energy density (u) [17]. In this study, a new method for gaining Young’s modulus (E) values with high accuracy extracted through the X-ray diffraction was introduced. For proving the practical parameters, NaCl powder was selected and calculation of this method was performed. Overall, the results were in good agreement with the experimental data and literature. Therefore, this new method of calculating Young’s modulus (E) values by X-ray diffraction is suggested for each single crystal or polycrystalline materials.

## 2. Materials and Experiments

In this study, a Bruker D8 Advance X-ray diffractometer with ${\mathrm{Cu}}_{K}{}_{\alpha }$ radiation was used. The powder X-ray diffraction was taken at 40 kV and 40 mA and recorded from 10 to 100 degrees for 2θ at a scanning speed of 2.5 degrees/min and a step size of 0.02 degrees. The XRD patterns were studied by High Score X’Pert software analysis and exported as ASC suffix files. Merck powder sodium chloride was prepared. A pulse echo method was used for the measurement of sound velocity for both transverse and longitudinal ultrasonic waves, and a specimen with a thickness of ~2 cm was fabricated. The model pulser receiver was Panametrics Co. (Waltham) and the oscilloscope was an Iwatsu (Japan) model (100 MHz). The resonant frequencies were considered as 10 and 5 MHz for the longitudinal and transverse waves, respectively. Furthermore, the three-dimensional (3D) geometry of crystal structures was designed by Crystal Maker, Version 10.2.2 software.

## 3. Methods

### 3.1. X-Ray Diffraction of Compound X

**Figure 1:**
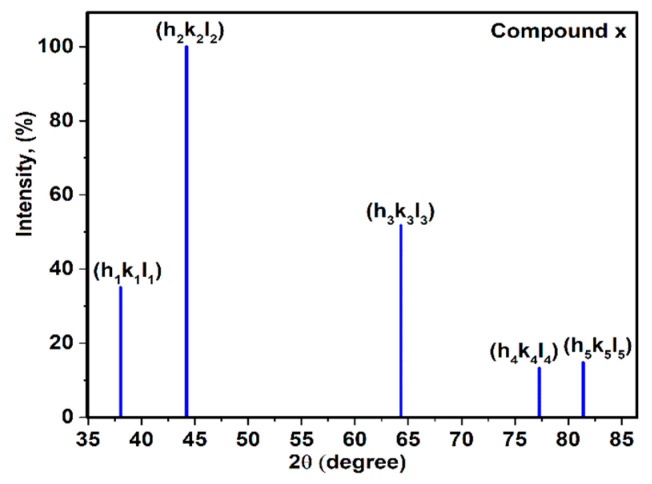
X-ray diffraction of compound x.

In this case, combining X-ray diffraction of crystalline materials with the planar density of each diffracted plane was performed. It is possible to accurately determine the Young’s modulus value of each crystalline solid material. The schematic XRD pattern was chosen for compound x (powder/crystal sample) (Figure 1). These crystallites are assumed to be randomly oriented to one another. In addition, if the powder is placed in the path of a monochromatic X-ray beam, diffraction will occur from the planes in those crystallites that are oriented at the correct angle to fulfill the Bragg condition. According to Figure 1, five planes consisting of ($h_{1}k_{1}l_{1}$), ($h_{2}k_{2}l_{2}$), ($h_{3}k_{3}l_{3}$), ($h_{4}k_{4}l_{4}$) and ($h_{5}k_{5}l_{5}$) were diffracted and diffracted beams were taken at an angle of 2ϴ for each plane with the incident beam. As a result extracted by the X’Pert software, the lattice parameter (a), index of planes (hkl) and lattice type (seven crystal systems) of the compound will be recognized. For following the gaining Young’s modulus value with high accuracy, the elastic stiffness constant values (C_ij_) and elastic compliance values (S_ij_) are needed.

### 3.2. Elastic Stiffness Constant and Elastic Compliance

**Figure 2:**
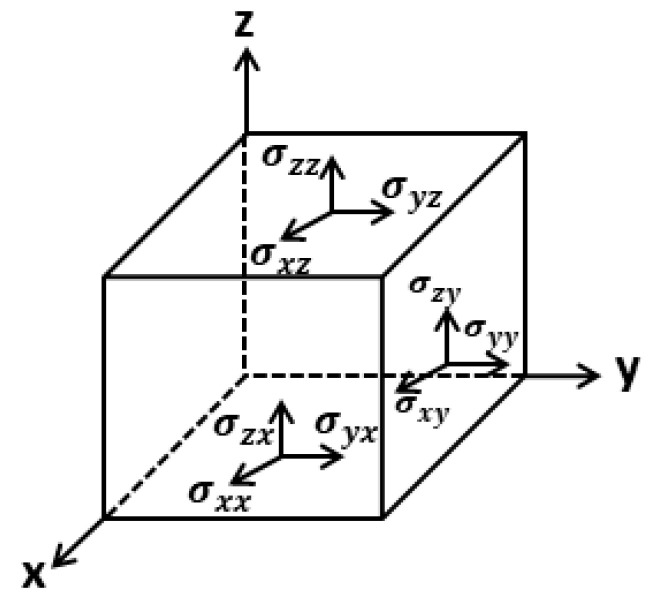
Elaborates of stress.

The stress components are specified as $\sigma_{\mathrm{ij}}$, where i is the direction of stress and j is the direction perpendicular to the plane upon which the stress is acting. The simple stress tensor is shown in Figure 2. Meanwhile, $\sigma_{\mathrm{xy}}$ is a stress in the x direction acting on the plane perpendicular to the y axis. Taking into account Figure 2, it reveals that there are nine stress components, however, since the presumption is that none are rotating, ($\sigma_{\mathrm{ij}}\;$= $\sigma_{\mathrm{ji}}$), the stress components are reduced to six: three axial, namely $\sigma_{\mathrm{xx}}$, $\sigma_{\mathrm{yy}}$ and $\sigma_{\mathrm{zz}}$, and three shear, namely $\sigma_{\mathrm{xy}}$, $\sigma_{\mathrm{yz}}$ and $\sigma_{\mathrm{xz}}$ [18].

According to the Equation (1) (Hooke’s law), the stress is proportional to the strain for small displacements. In the generalized form, this proportionality principle is extended to the six stresses and strains [19].

(1) $$E\;=\;\frac{\sigma }{\varepsilon }$$

Nevertheless, Hooke’s law can be written such as:

$\sigma_{\mathrm{xx}}=\;C_{11}\varepsilon_{\mathrm{xx}}$ + $C_{12}\varepsilon_{\mathrm{yy}}$ + $C_{13}\varepsilon_{\mathrm{zz}}$ + $C_{14}\varepsilon_{\mathrm{yz}}$ + $C_{15}\varepsilon_{\mathrm{zx}}$ + $C_{16}\varepsilon_{\mathrm{xy}}$

$\sigma_{\mathrm{yy}}=\;C_{21}\varepsilon_{\mathrm{xx}}$ + $C_{22}\varepsilon_{\mathrm{yy}}$ + $C_{23}\varepsilon_{\mathrm{zz}}$ + $C_{24}\varepsilon_{\mathrm{yz}}$ + $C_{25}\varepsilon_{\mathrm{zx}}$ + $C_{26}\varepsilon_{\mathrm{xy}}$

$\sigma_{\mathrm{zz}}=\;C_{31}\varepsilon_{\mathrm{xx}}$ + $C_{32}\varepsilon_{\mathrm{yy}}$ + $C_{33}\varepsilon_{\mathrm{zz}}$ + $C_{34}\varepsilon_{\mathrm{yz}}$ + $C_{35}\varepsilon_{\mathrm{zx}}$ + $C_{36}\varepsilon_{\mathrm{xy}}$

$\sigma_{\mathrm{yz}}=\;C_{41}\varepsilon_{\mathrm{xx}}$ + $C_{42}\varepsilon_{\mathrm{yy}}$ + $C_{43}\varepsilon_{\mathrm{zz}}$ + $C_{44}\varepsilon_{\mathrm{yz}}$ + $C_{45}\varepsilon_{\mathrm{zx}}$+ $C_{46}\varepsilon_{\mathrm{xy}}$

$\sigma_{\mathrm{zx}}=\;C_{51}\varepsilon_{\mathrm{xx}}$ + $C_{52}\varepsilon_{\mathrm{yy}}$ + $C_{53}\varepsilon_{\mathrm{zz}}$ + $C_{54}\varepsilon_{\mathrm{yz}}$ + $C_{55}\varepsilon_{\mathrm{zx}}$ + $C_{56}\varepsilon_{\mathrm{xy}}$

$\sigma_{\mathrm{xy}}=\;C_{61}\varepsilon_{\mathrm{xx}}$ + $C_{62}\varepsilon_{\mathrm{yy}}$ + $C_{63}\varepsilon_{\mathrm{zz}}$ + $C_{64}\varepsilon_{\mathrm{yz}}$ + $C_{65}\varepsilon_{\mathrm{zx}}$ + $C_{66}\varepsilon_{\mathrm{xy}}$

The constants of proportionality $C_{\mathrm{ij}}$ are introduced as the elastic constants. Since for a small displacement the elastic energy density is a quadratic function of the strains, and the elastic constants are given by the second partial derivatives of the energy density, it can be shown that $C_{\mathrm{ij}}$= $C_{\mathrm{ji}}$. Meanwhile, the 36 elastic constants result in the matrix form and they can be decreased to 6 diagonal components and 15 off-diagonal elaborates. In addition, it is possible for each system of crystal structures that the number of matrices can be decreased. According to Equation (2) in matrix form, these elastic constants can be written as [20,21]

(2) $$\left|\begin{array}{cccccc} C_{11} & C_{12} & C_{13} & C_{14} & C_{15} & C_{16}\\ C_{21} & C_{22} & C_{23} & C_{24} & C_{25} & C_{26}\\ C_{31} & C_{32} & C_{33} & C_{34} & C_{35} & C_{36}\\ C_{41} & C_{42} & C_{43} & C_{44} & C_{45} & C_{46}\\ C_{51} & C_{52} & C_{53} & C_{54} & C_{55} & C_{56}\\ C_{61} & C_{62} & C_{63} & C_{64} & C_{65} & C_{66} \end{array}\right|$$

The specified form of equation 1 can be written in decreased tensor notation and the form is the so-called Voigt notation (Equation (3)).

(3) $$\sigma_{i}={\;C}_{\mathrm{ij}}\varepsilon_{j}$$

i and j equal 1 to 6, and i, j, which goes from 1 through 6, is related to xx, yy, zz, yz, zx and xy, respectively.

Furthermore, in the practical subjects, it is preferred to take strains into account in terms of the stresses. According to Equation (3), Equation (4) can be written

(4) $$\varepsilon_{i}={\;S}_{\mathrm{ij}}\sigma_{j}$$

Here, $S_{\mathrm{ij}}$ is introduced as the elastic compliance constants, so the exact formula can be written as the below:

$\varepsilon_{1}=\;S_{11}\sigma_{1}$ + $S_{12}\sigma_{2}$ + $S_{13}\sigma_{3}$ + $S_{14}\sigma_{4}$ + $S_{15}\sigma_{5}$ + $S_{16}\sigma_{6}$

$\varepsilon_{2}=\;S_{21}\sigma_{1}$ + $S_{22}\sigma_{2}$ + $S_{23}\sigma_{3}$ + $S_{24}\sigma_{4}$ + $S_{25}\sigma_{5}$ + $S_{26}\sigma_{6}$

$\varepsilon_{3}=\;S_{31}\sigma_{1}$ + $S_{32}\sigma_{2}$ + $S_{33}\sigma_{3}$ + $S_{34}\sigma_{4}$ + $S_{35}\sigma_{5}$ + $S_{36}\sigma_{6}$

$\varepsilon_{4}=\;S_{41}\sigma_{1}$ + $S_{42}\sigma_{2}$ + $S_{43}\sigma_{3}$ + $S_{44}\sigma_{4}$ + $S_{45}\sigma_{5}$ + $S_{46}\sigma_{6}$

$\varepsilon_{5}=\;S_{51}\sigma_{1}$ + $S_{52}\sigma_{2}$ + $S_{53}\sigma_{3}$ + $S_{54}\sigma_{4}$ + $S_{55}\sigma_{5}$ + $S_{56}\sigma_{6}$

$\varepsilon_{6}=\;S_{61}\sigma_{1}$ + $S_{62}\sigma_{2}$ + $S_{63}\sigma_{3}$ + $S_{64}\sigma_{4}$ + $S_{65}\sigma_{5}$ + $S_{66}\sigma_{6}$

There are seven conventional unit cells of Bravais lattices consisting of triclinic, monoclinic, orthorhombic, tetragonal, cubic, trigonal and hexagonal. Moreover, each lattice has special elastic stiffness constant values and elastic compliance values. The triclinic matrix has 21 elastic constants and it is the conventional wisdom. However, Landau and Lifshitz suggested that this number should be decreased by 3 to 18 due to the choice of the Cartesian orthogonal axes x, y and z, and it is arbitrary and could be interchanged, therefore decreasing the number by three [22].

Triclinic matrix $\left|\begin{array}{cccccc} C_{11} & C_{12} & C_{13} & C_{14} & C_{15} & C_{16}\\ C_{21} & C_{22} & C_{23} & C_{24} & C_{25} & C_{26}\\ C_{31} & C_{32} & C_{33} & C_{34} & C_{35} & C_{36}\\ C_{41} & C_{42} & C_{43} & C_{44} & C_{45} & C_{46}\\ C_{51} & C_{52} & C_{53} & C_{54} & C_{55} & C_{56}\\ C_{61} & C_{62} & C_{63} & C_{64} & C_{65} & C_{66} \end{array}\right|$

Monoclinic has 13 elastic constants and due to the suitable selection of the coordinate axes, these should be decreased to 12.

Monoclinic matrix $\left|\begin{array}{cccccc} C_{11} & C_{12} & C_{13} & 0 & C_{15} & 0\\ C_{21} & C_{22} & C_{23} & 0 & C_{25} & 0\\ C_{31} & C_{32} & C_{33} & 0 & C_{35} & 0\\ 0 & 0 & 0 & C_{44} & 0 & C_{46}\\ C_{51} & C_{52} & C_{53} & 0 & C_{55} & 0\\ 0 & 0 & 0 & C_{64} & 0 & C_{66} \end{array}\right|$

Orthorhombic elastic constants are obtained from nine components.

Orthorhombic matrix $\left|\begin{array}{cccccc} C_{11} & C_{12} & C_{13} & 0 & 0 & 0\\ C_{21} & C_{22} & C_{23} & 0 & 0 & 0\\ C_{31} & C_{32} & C_{33} & 0 & 0 & 0\\ 0 & 0 & 0 & C_{44} & 0 & 0\\ 0 & 0 & 0 & 0 & C_{55} & 0\\ 0 & 0 & 0 & 0 & 0 & C_{66} \end{array}\right|$

Trigonal has two types of elastic constants: the first one is for the symmetry groups that have seven constants and the second one is for the suitable selection of coordinates, and these should be decreased to six constants.

Trigonal matrix (1) $\left|\begin{array}{cccccc} C_{11} & C_{12} & C_{13} & C_{14} & -C_{25} & 0\\ C_{12} & C_{11} & C_{13} & -C_{14} & C_{25} & 0\\ C_{13} & C_{13} & C_{33} & 0 & 0 & 0\\ C_{14} & -C_{14} & 0 & C_{44} & 0 & C_{25}\\ -C_{25} & C_{25} & 0 & 0 & C_{44} & C_{14}\\ 0 & 0 & 0 & C_{25} & C_{14} & \frac{1}{2}\left(C_{11}-C_{12}\right) \end{array}\right|$

Trigonal matrix (2) $\left|\begin{array}{cccccc} C_{11} & C_{12} & C_{13} & C_{14} & 0 & 0\\ C_{12} & C_{11} & C_{13} & -C_{14} & 0 & 0\\ C_{13} & C_{13} & C_{33} & 0 & 0 & 0\\ C_{14} & -C_{14} & 0 & C_{44} & 0 & C_{25}\\ -C_{25} & C_{25} & 0 & 0 & C_{44} & C_{14}\\ 0 & 0 & 0 & 0 & C_{14} & \frac{1}{2}\left(C_{11}-C_{12}\right) \end{array}\right|$

The tetragonal system has two types of elastic constants as well: the first one is for the symmetry groups that have seven constants and the second one is for the suitable selection of coordinates, and these should be decreased to six constants [21,23].

Tetragonal matrix (1) $\left|\begin{array}{cccccc} C_{11} & C_{12} & C_{13} & 0 & 0 & C_{16}\\ C_{12} & C_{11} & C_{13} & 0 & 0 & -C_{16}\\ C_{13} & C_{13} & C_{33} & 0 & 0 & 0\\ 0 & 0 & 0 & C_{44} & 0 & 0\\ 0 & 0 & 0 & 0 & C_{44} & 0\\ C_{16} & -C_{16} & 0 & 0 & 0 & C_{66} \end{array}\right|$

Tetragonal matrix (2) $\left|\begin{array}{cccccc} C_{11} & C_{12} & C_{13} & 0 & 0 & 0\\ C_{12} & C_{11} & C_{13} & 0 & 0 & 0\\ C_{13} & C_{13} & C_{33} & 0 & 0 & 0\\ 0 & 0 & 0 & C_{44} & 0 & 0\\ 0 & 0 & 0 & 0 & C_{44} & 0\\ 0 & 0 & 0 & 0 & 0 & C_{66} \end{array}\right|$

The hexagonal system has five elastic constants.

Hexagonal matrix $\left|\begin{array}{cccccc} C_{11} & C_{12} & C_{13} & 0 & 0 & 0\\ C_{12} & C_{11} & C_{13} & 0 & 0 & 0\\ C_{13} & C_{13} & C_{33} & 0 & 0 & 0\\ 0 & 0 & 0 & C_{44} & 0 & 0\\ 0 & 0 & 0 & 0 & C_{44} & 0\\ 0 & 0 & 0 & 0 & 0 & \frac{1}{2}\left(C_{11}-C_{12}\right) \end{array}\right|$

The cubic system has three elastic constants.

Cubic matrix $\left|\begin{array}{cccccc} C_{11} & C_{12} & C_{13} & 0 & 0 & 0\\ C_{12} & C_{11} & C_{13} & 0 & 0 & 0\\ C_{13} & C_{13} & C_{33} & 0 & 0 & 0\\ 0 & 0 & 0 & C_{44} & 0 & 0\\ 0 & 0 & 0 & 0 & C_{44} & 0\\ 0 & 0 & 0 & 0 & 0 & C_{44} \end{array}\right|$

For conventional systems consisting of cubic and hexagonal structures, the relationships between $C_{\mathrm{ij}}$ and $S_{\mathrm{ij}}$ are presented in Equations (5)–(12), respectively.

For cubic [24,25]:

(5) $$S_{11}=\;\frac{C_{11}+C_{12}}{\left(C_{11}-C_{12}\right)\left(C_{11}+2C_{12}\right)}$$

(6) $$S_{12}=\;\frac{-C_{12}}{\left(C_{11}-C_{12}\right)\left(C_{11}+2C_{12}\right)}$$

(7) $$S_{44}=\;\frac{1}{C_{44}}$$

For hexagonal [24,26]:

(8) $$S_{11}=\frac{1}{2}\;\left(\frac{C_{33}}{C_{33}\left(C_{11}+C_{12}\right)-2{\left(C_{13}\right)}^{2}}+\;\frac{1}{C_{11}-C_{12}}\right)$$

(9) $$S_{12}=\frac{1}{2}\;\left(\frac{C_{33}}{C_{33}\left(C_{11}+C_{12}\right)-2{\left(C_{13}\right)}^{2}}-\;\frac{1}{C_{11}-C_{12}}\right)$$

(10) $$S_{33}=\frac{C_{11}+C_{12}}{C_{33}\left(C_{11}+C_{12}\right)-2{\left(C_{13}\right)}^{2}}$$

(11) $$S_{13}=-\frac{C_{13}}{C_{33}\left(C_{11}+C_{12}\right)-2{\left(C_{13}\right)}^{2}}$$

(12) $$S_{44}=\frac{1}{C_{44}}$$

Mostly, elastic constant values of materials are tabulated in the literature [25,26]. Furthermore, the Young’s modulus of each plane (E_hkl_) can be expressed for cubic and hexagonal crystals respectively as Equations (13) and (14).

For cubic [27]:

(13) $$\frac{1}{E_{\mathrm{hkl}}}\;=\;S_{11}-\;2\left[\left(S_{11}-S_{12}\right)-\frac{1}{2}S_{44}\right]\left[\frac{h^{2}k^{2}+{\;k}^{2}l^{2}+{\;l}^{2}h^{2}}{\left(h^{2}+{\;k}^{2}+{\;l}^{2}\right)}\right]$$

For hexagonal [28,29]:

(14) $$E_{\mathrm{hkl}}=\;\frac{{\left[h^{2}+\frac{{\left(h+2k\right)}^{2}}{3}+{\left(\frac{\mathrm{al}}{c}\right)}^{2}\right]}^{2}}{S_{11}{\left(h^{2}+\frac{{\left(h+2k\right)}^{2}}{3}\right)}^{2}+S_{33}{\left(\frac{\mathrm{al}}{c}\right)}^{4}+\left(2S_{13}+S_{44}\right)\left(h^{2}+\frac{{\left(h+2k\right)}^{2}}{3}\right){\left(\frac{\mathrm{al}}{c}\right)}^{2}}$$

### 3.3. Relationship between Young’s Modulus E_hkl_ and Planar Density of Each Diffracted Plane through X-Ray Diffraction

**Figure 3:**
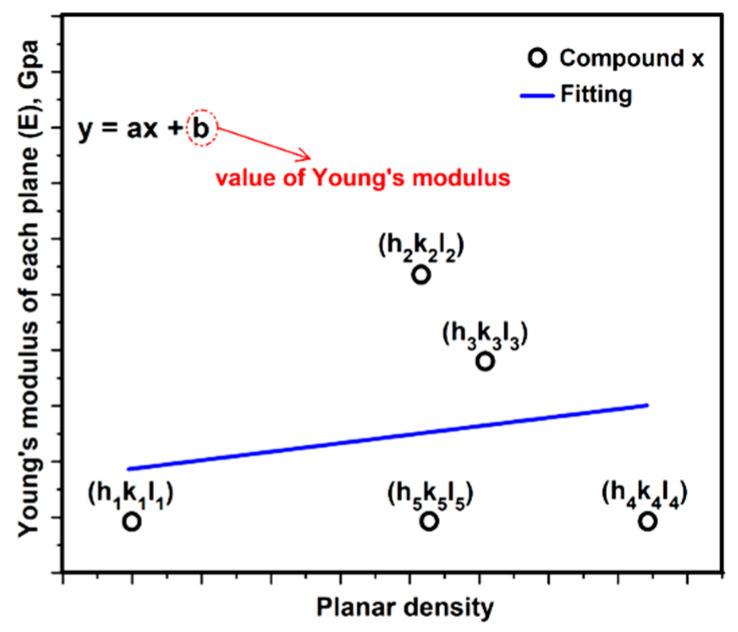
Young’s modulus extracted from planes of compound x versus planar density.

The type of crystal lattice of compound x must be determined according to the X-ray diffraction file. In this case, it is presumed that compound x has a cubic crystal structure. According to Equations (5)–(7), elastic compliance values of compound x were calculated as the $S_{11}$, $S_{12}$ and $S_{44}$, therefore, Young’s modulus values of diffracted planes (Figure 1) were registered by Equation (13). The values of Young’s modulus of diffracted planes or modulus of elasticity (E) of compound x are named $E_{\left(h_{1}k_{1}l_{1}\right)\;},\;E_{\left(h_{2}k_{2}l_{2}\right)\;},\;E_{\left(h_{3}k_{3}l_{3}\right)\;},\;E_{\left(h_{4}k_{4}l_{4}\right)}$ and $E_{\left(h_{5}k_{5}l_{5}\right)}$. In addition, planar density (PD) values of diffracted planes were calculated as ${\mathrm{PD}}_{\left(h_{1}k_{1}l_{1}\right)\;},\;{\mathrm{PD}}_{\left(h_{2}k_{2}l_{2}\right)\;},\;{\mathrm{PD}}_{\left(h_{3}k_{3}l_{3}\right)\;},\;{\mathrm{PD}}_{\left(h_{4}k_{4}l_{4}\right)}$ and ${\mathrm{PD}}_{\left(h_{5}k_{5}l_{5}\right)}$. Applying the least squares method for the line between the values extracted of Young’s modulus and planar density of diffracted planes can give the average Young’s modulus value of compound x with high accuracy. In this method, Young’s modulus values of diffracted planes play a role as the y axis, and the x axis is the planar density. The Young’s modules value extracted from each plane of compound x versus the planar density is presented in Figure 3.

In this method, the unit of Young’s modulus value is dependent on the unit of the elastic stiffness constant and elastic compliance values, and here it is GPa. Moreover, planar density does not always have a unit and the value of planar density is always less than 1. In addition, the values of planar density are dependent on the situation of the planes. For example, in this curve (Figure 3) for compound x, ${\mathrm{PD}}_{\left(h_{4}k_{4}l_{4}\right)}$ > ${\mathrm{PD}}_{\left(h_{3}k_{3}l_{3}\right)}$ > ${\mathrm{PD}}_{\left(h_{5}k_{5}l_{5}\right)}$ > ${\mathrm{PD}}_{\left(h_{2}k_{2}l_{2}\right)}$ > ${\mathrm{PD}}_{\left(h_{1}k_{1}l_{1}\right)}.$ Furthermore, it is possible that two or more planes have similar planar density values.

In this method, the empty planes in one unit cell are not considered in the calculations. However, if there is no atom in the plane inside the unit cell, they will appear when the plane is extended to the adjacent cells and atoms will appear and the unit cell will be converted to super cells, and calculating the planar density for two or more adjacent unit cells or super cells is possible.

**Figure 4:**
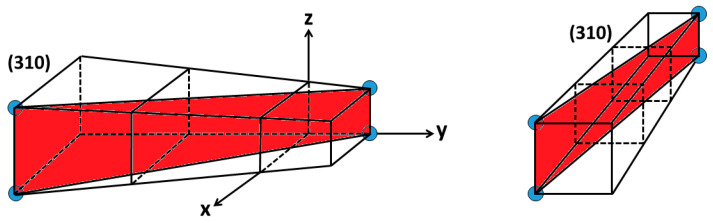
Schematics of (310) expanded in three unit cells.

In some cases, such as plane (310) in simple cubic, as observed in Figure 4, there are two corner atoms in the original crystal lattice. However, when the unit cell is extended, there is no atom in the adjacent crystal. In this case, the planar density should be calculated taking into account the three adjacent crystal lattices. In other words, when the (310) plane is continued and extended to three unit cells, there is no atom in the middle one, but there are 2 ×$\;\frac{1}{2}$ atoms in the first and third unit cells.

When atoms are compacted, a higher modulus of elasticity is obtained. For example, for Fe (BCC), $E_{\left(100\right)}$ is equal to 125 GPa and $E_{\left(111\right)}$ is equal to 272.7 GPa [30], since the density in the (111) plane of Fe (BCC) is higher and needs more force for the displacement of atoms. Furthermore, controlling the process of deformation and displacement of atoms in the planes is related to the dislocation networks. The force (W) for moving atoms in each plane corresponds to Equation (15) [31,32].

(15) W = Gb^2^l

where G is the shear modulus, b the Burgers vector and l is the dislocation length. In addition, substitution of G with Equation (16) can be provided for gaining Equation (17). The E is Young’s modulus and ν is Poisson’s ratio.

(16) $$G\;=\;\frac{E}{2\left(1+\nu \right)}$$

(17) $$W\;=\;\frac{E}{2\left(1+\nu \right)}\;b^{2}l$$

**Figure 5:**
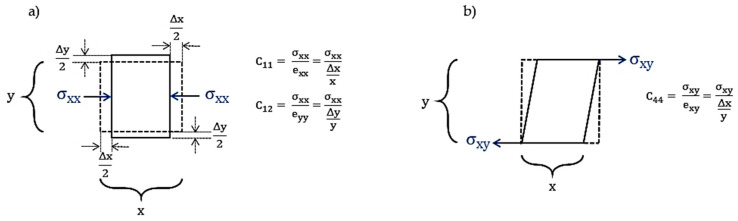
(**a**) Longitudinal compression $C_{11}$ and transverse expansion $C_{12}$; (**b**) shear modulus $C_{44}$ [39].

When atoms are at far distances, such as corner atoms in (100), atoms need higher values of applied force for approaching [33,34]. Active slip systems, a combination of the slip plane and slip direction, are required for plastic deformation [35,36,37]. Moreover, dislocation interactions can be the result [38]. X-ray diffraction provides information about the diffracted phase and the position of atoms in the phase. In this research, the density of atoms in any plane is introduced as the phase density, which is defined as the area of the atoms with the center located at the plane, divided by the total area of the plane, and it is responsible for the mechanical properties of each plane. The fundamental assumption is that when the density of a plane is increased, the movement of atoms with the mechanism of dislocation motion requires high forces. The increase in the force means that the modulus of elasticity (Young’s modulus) would be a higher value. Now, we consider the modulus of elasticity for each phase with a specific phase density, where the useful information is obtained by plotting the Young’s modulus versus the planar density, by the least squares method. Decreasing the sources of error by segregation of data provides the best phase and most accurate intercept. The intercept is responsible for the average Young’s modulus of materials, from which mechanical properties of different materials can be specified. In the other aspect, $C_{11}$ is in agreement with the longitudinal distortion and longitudinal compression/tension, thus $C_{11}$ can be described as the hardness. Moreover, the transverse distortion is related to $C_{12}$, and $C_{12}$ is derived from the transverse expansion corresponding to the Poisson’s ratio. In addition, $C_{44}$ is based on the shear modulus, and further, $C_{44}$ is in the settlement with $C_{11}$ and $C_{12}$ [24,39]. The schematic role of elastic constants in cubic structures is presented in Figure 5 [39]. Accordingly, the shear modulus is in a relationship with the Burgers vector and Young’s modulus (Equations (16) and (17)), and the dislocation density is in agreement with Young’s modulus through Equations (18) and (19) [40,41].

(18) $$\rho \;=\;\frac{E}{\vdots E}$$

where E is Young’s modulus and ⋮E is introduced as Equation (19).

(19) $$\vdots E\;=\;\frac{{\mathrm{Gb}}^{2}}{2}$$

Nevertheless, the least squares technique can be applied for recording the Young’s modulus and planar density values of each diffracted plane via X-ray diffraction. In addition, this method can be utilized for modifying the Williamson–Hall (W–H) method in the uniform stress deformation model (USDM) due to minimizing the sources of errors.

### 3.4. Modified W–H (USDM Model)

The W–H method uses the full width at half maximum (FWHM) of the diffraction peaks for determining different elastic properties. We must decrease the errors mathematically and obtain the average value of E through all the peaks (or diffracted peaks) by using the least squares method. According to the physical broadening ($\beta_{\mathrm{hkl}}$) of the X-ray diffraction peak, it is a combination of size and strain. The W–H method is a simplified integral expanse where strain-induced broadening is distinguished through taking into account the peak width [42]. In the W–H method through the USDM model, Young’s modulus values are considered. According to Equation (20), terms of $\frac{4\sin\theta }{E_{\mathrm{hkl}}}$ along the *X*-axis and $\beta_{\mathrm{hkl}}.\cos\theta$ along the *Y*-axis are related to the XRD pattern of samples.

(20) $$\beta_{\mathrm{hkl}}.\cos\theta \;=\;(\frac{K\lambda }{L})\;+\;4\sigma .\frac{\sin\theta }{E_{\mathrm{hkl}}}$$

$\beta_{\mathrm{hkl}}$ introduced the broadening of the peak derived from the (hkl) plane. In this model, calculating the strain and average Young’s modulus values is performed. Until now, the average E value was calculated in research and studies by utilizing the W–H method, but it is with errors because when the average values of E were considered, the final value of E would be far from the standard value of E in each peak extracted by X-ray diffraction. Furthermore, in some studies, the E value is considered as a value tabulated for material, but this value is not real. For illustration, based on studies of Ref [43], the value of E for cadmium selenide (CdSe) is calculated as an average value and E is reported as ~79.233 GPa, so this average value is very far from the standard E value of CdSe that is reported in Ref [44,45]. The E value for zinc oxide (ZnO) through the USDM model is reported as~130 GPa and this value is different from other studies of E_ZnO_ related to this material such as Ref [46,47]. In another study, the USDM model was used for yttrium oxide (Y_2_O_3_) [48] and the reported E value did not correspond to other studies such as Ref [49,50]. In this case, the elastic constant values of sodium chloride (NaCl) were measured via resonant-ultrasound spectroscopy similar to Ref [51,52]. The experimental and theoretical elastic constants and elastic compliance constants in previous research and this study are reported in Table 1 and Table 2, respectively.

**Figure 6:**
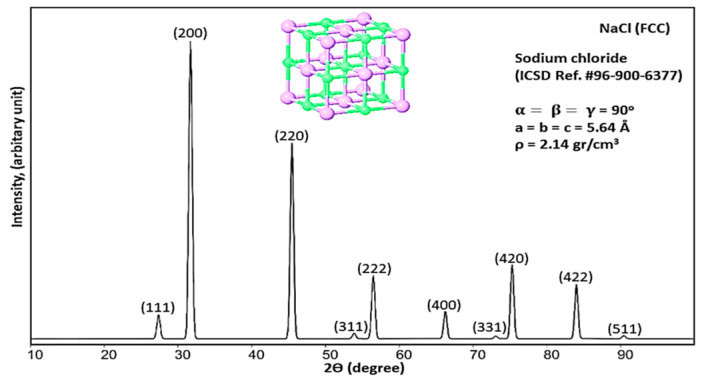
X-ray diffraction of NaCl powder sample.

The X-ray diffraction of NaCl is presented in Figure 6. The characterization peaks of NaCl are very close to the corresponding report in Ref [57]. NaCl has ionic bonds, and the ionic radius of Na^+^ and Cl^−^ is 0.97 and 1.81 Å, respectively. The crystal of NaCl is FCC, and the location of the atom of Cl introduces (000) and Na at ($\frac{1}{2}\;\frac{1}{2}\;\frac{1}{2}$) position. According to the X’Pert analysis, the lattice parameter has gained 5.640 Å and it is in good agreement with the values reported in Ref [58]. In addition, crystallographic parameters of NaCl resulting from X’Pert are submitted in Table 3.

**Figure 7:**
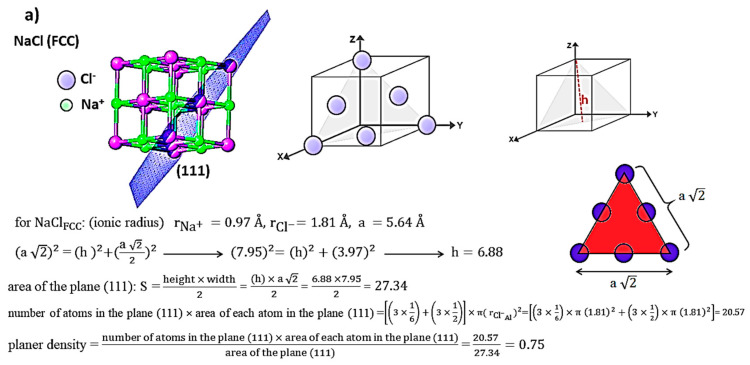

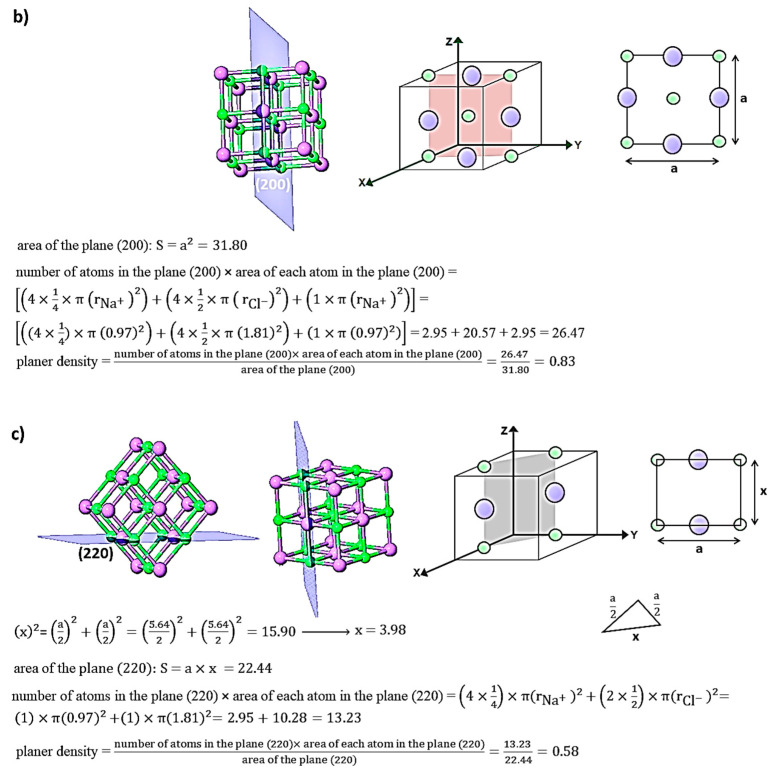
Geometry and the situation of involved atoms in diffracted planes (**a**) (111), (**b**) (200) and (**c**) (220).

According to Figure 6, ten planes have shown diffraction. According to the discussed method in this study for gaining planar density values of each diffracted plane, the schematic geometry of planes, the situation of diffracted planes and the location of atoms in the diffracted planes in the unit cell are shown. The situation of diffracted planes and atoms of NaCl and the calculation of the planar density values of each diffracted plane are presented in Figure 7 and Figure S1.

**Figure 8:**
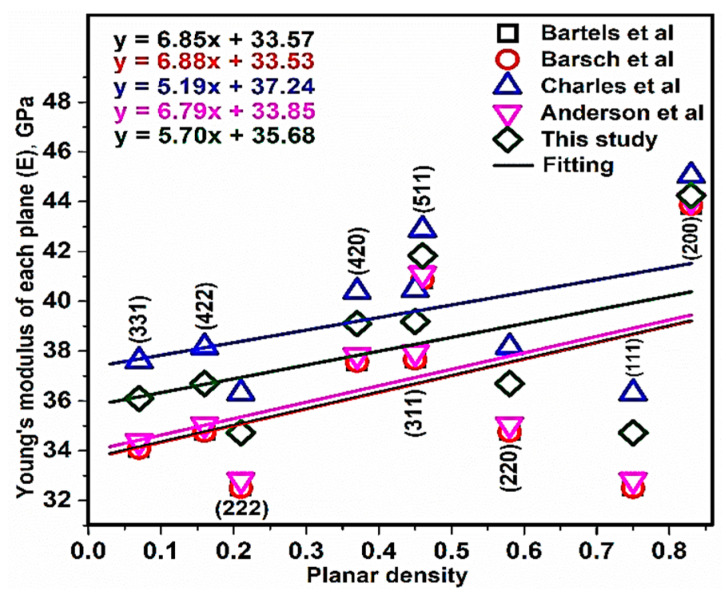
Young’s modulus of each plane of NaCl extracted by XRD patterns versus planar density.

According to Equation (13) (because NaCl has an FCC structure), the values of elastic compliances (S) (Table 2) were substituted and $E_{\mathrm{hkl}}$ values of each diffrected plane were calculated as 34.72, 44.24, 36.69, 39.18, 34.72, 36.10, 39.09, 36.69 and 41.83 GPa for planes (111), (200), (220), (311), (222), (331), (420), (422) and (511), respectively. In addition, there is no atom in (400) (discussed in the previous part), therefore this plane is not considered in the calculations. The Young’s modulus of each plane of NaCl extracted by XRD patterns versus planar density is presented in Figure 8. Linear equations of NaCl recorded y = 6.85x + 33.57 (Bartels et al.), y = 6.88x + 33.53 (Barsch et al.), y = 5.19x + 37.24 (Charles et al.), y = 6.79x + 33.85 (Anderson et al.) and y = 5.70x + 35.68 (this study). Nevertheless, the intercepts were calculated as 33.57, 33.53, 37.24, 33.85 and 35.68 GPa, respectively, and the values of intercepts (Young’s modulus) are submitted in Table 4.

For evaluation of this method, the ultrasonic technique was used for measuring Young’s modulus values of NaCl. Ultrasonic wave is a kind of elastic wave propagating in the medium with a higher frequency of gaining the Young’s modulus value of samples. In the ultrasonic method, longitudinal and transverse waves were used for measuring Young’s modulus value [59,60]. In this technique, by measuring the velocity of ultrasound waves and the density of the specimen, the calculation of Young’s modulus value is performed (Equation (21)).

(21) $$E\;=\;\frac{{\rho c}_{l}^{2}[3\left(\frac{c_{l}}{c_{t}}{)}^{2}-4\right]}{{\left(\frac{c_{l}}{c_{t}}\right)}^{2}-1}$$

In this equation, ρ, $c_{l}$ and $c_{t}$ are the density, and the velocity of ultrasound longitudinal and transverse waves, respectively. According to the differences between two echoes (t = t_2_ − t_1_) in the signals and knowing the length of the specimen, the velocity of ultrasound longitudinal and transverse waves can be calculated through Equation (22).

(22) $$c\;=\;\frac{2L}{t}$$

where L is the length of the specimen and t is the differences between two echoes. The density of the specimen can be registered via measuring the mass and volume of the sample [61]. In addition, with placing Equation (22) in Equation (21), the main formula for calculating the Young’s modulus value is introduced as Equation (23).

(23) $$E\;=\;\frac{4\rho {\left(\frac{L}{t_{s}}\right)}^{2}\left(3t_{s}^{2}-4t_{l}^{2}\right)}{t_{s}^{2}-t_{l}^{2}}$$

where $t_{s}$ and $t_{l}$ are differences between two echoes in longitudinal and transverse waves, respectively [61]. After measuring five times for adjustments of longitudinal and transverse waves, the Young’s modulus value is gained as 34.83 GPa. The value of Young’s modulus extracted through the ultrasonic technique was in good agreement with the value extracted in this study (Table 4).

### 3.5. Modified Williamson–Hall Method (USDM) for NaCl

**Figure 9:**
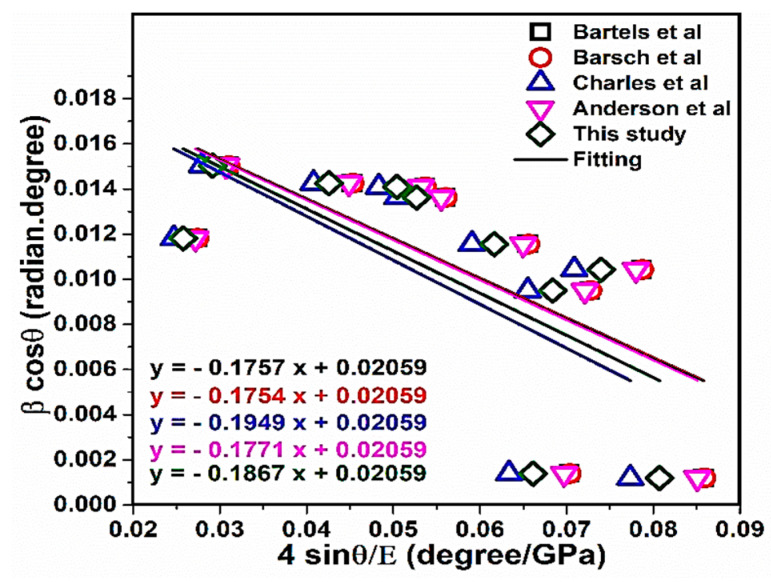
Modified (William–Hall, W–H) uniform stress deformation model (USDM) plot of NaCl.

For a more realistic NaCl crystal system, the anisotropic nature of Young’s modulus is considered [62]. The generalization of Hook’s law (Equation (1)) is that the strain (ε) and stress (𝜎) are in a linear relationship with the constant of proportionality, being the modulus of elasticity or simply Young’s modulus. In this method, for the NaCl structure, Hooke’s law was performed for strain and stress, taking into account the linear proportionality of Equation (20). This equation is just an access that it is credible for a small strain notably. Furthermore, increasing the strain will cause the deviation of atoms from the linear relationship [63]. The exact parameters resulting from X-ray diffraction are given in Table 5. According to the W–H method in the USDM model, $\frac{4\;\sin\theta }{E}$ (degree/GPa) played a role as the X axis and β (radian).cosθ (degree) played a role as the Y axis (Figure 9). According to Figure 9, the slope values are related to the stress (σ). Values of stress and strain have gained negative values. The positive values of intrinsic strain and stress can be provided the tensile strain and stress, and if values are negative, such as Table 5, they will be related to the compressive stress and strain. Moreover, the stress (σ) and strain (ε) values extracted by the modified W–H method in the USDM model, shear modulus (µ), Poisson’s ratio (υ) and bulk modulus (B) of NaCl are reported in Table 6. The values of mechanical properties (Table 6) were in good agreement with the values tabulated in Ref [64].

## 4. Conclusions

The clear, short and frank conclusions are as follows:

1. A new method for measuring the accurate value of the modulus of elasticity of crystalline materials is successfully presented.
2. Planar density for the area of total atoms/ions in the plane divided by the plane area is responsible for the modulus of elasticity of that plane.
3. Modulus of elasticity of each plane (y axis) is plotted against the planar density of that plane (x axis), by the least squares method, to give the Young’s modulus of the materials at the intercept.
4. Case study of NaCl proved the accuracy of the new method in this study, in good agreement with the ultrasonic technique.
5. The Williamson–Hall method, especially in the uniform stress deformation model (USDM), can be used in this method to minimize errors in the least squares method and yield a proper modulus of elasticity, much more accurate than the average value.
6. The restriction is that XRD data for planar density calculations are applicable in the uniform distribution of atoms in the crystal lattice with a unit cell, so the method cannot be used for amorphous materials.
7. This method can be applied for research as well as industrial applications.

## Figures and Tables

**Table 1:** Elastic constant values of NaCl extracted by experimental/theoretical literature and this study.

Elastic Constant (C), (Gpa)	Expt. from Ref (Bartels et al.) [53]	Expt. from Ref (Barsch et al.) [54]	Expt. from Ref (Charles et al.) [55]	Theory. from Ref (Anderson et al.) [56]	This Study
**C_11_**	48.99	49.00	50.00	49.50	49.11
**C_12_**	12.57	12.60	12.70	13.20	12.26
**C_44_**	12.72	12.70	14.40	12.79	13.73

**Table 2:** Elastic compliance values of NaCl extracted by experimental/theoretical literature and this study.

Elastic Compliances (S), (Gpa)	Expt. by (Bartels et al.)	Expt. by (Barsch et al.)	Expt. by (Charles et al.)	Theory. by (Anderson et al.)	This Study
**S_11_**	0.0228	0.0228	0.0222	0.0227	0.0226
**S_12_**	−0.0046	−0.0046	−0.0045	−0.0047	−0.0045
**S_44_**	0.0786	0.0787	0.0694	0.0781	0.0728

**Table 3:** Crystallographic parameters of the NaCl (FCC) structure resulting from the X’Pert software.

NaCl					
**Crystal System**	a ( Å)	c ( Å)	Cell Volume $(Å{)}^{3}$	Crystal Density (g/cm^3^)	Space Group
FCC	5.640	5.640	181.511	2.141	Fm-3m

**Table 4:** Young’s modulus values of NaCl.

Study	Young Modulus (E), (Gpa) in This Method (Intercept Value)
**Expt. by (Bartels et al.)**	33.57
**Expt. by (Barsch et al.)**	33.53
**Expt. by (Charles et al.)**	37.24
**Theory. by (Anderson et al.)**	33.85
**This study**	35.68

**Table 5:** Crystallographic parameters of each individual XRD pattern related to NaCl.

NaCl										
2θ (Degree)	Β = FWHM (Degree)	θ (Degree)	cosθ (Degree)	1/cosθ (Degree)	Ln(1/cosθ) (Degree)	Β = FWHM (Radian)	Ln β (Radian)	4 sinθ (Degree)	β(Radian).cosθ (Degree)	hkl
27.01	0.70	13.50	0.97	1.03093	0.03046	0.01218	−4.40796	0.92	0.01181	111
30.91	0.90	15.45	0.96	1.04167	0.04082	0.01566	−4.15665	1.04	0.01503	200
45.08	0.89	22.54	0.92	1.08696	0.08338	0.01549	−4.16782	1.52	0.01425	220
53.70	0.91	26.85	0.89	1.1236	0.11653	0.01583	−4.1456	1.80	0.01409	311
56.79	0.90	28.39	0.87	1.14943	0.13926	0.01566	−4.15665	1.88	0.01362	222
66.98	0.80	33.49	0.83	1.20482	0.18633	0.01392	−4.27443	2.20	0.01155	400
72.99	0.10	36.49	0.80	1.25	0.22314	0.00174	−6.35387	2.36	0.00139	331
76.05	0.70	38.02	0.78	1.28205	0.24846	0.01218	−4.40796	2.44	0.0095	420
83.93	0.81	41.96	0.74	1.35135	0.30111	0.01409	−4.26201	2.64	0.01043	422
92.60	0.10	46.30	0.69	1.44928	0.37106	0.00174	−6.35387	2.88	0.0012	511

**Table 6:** Values derived from the mechanical properties related to NaCl.

Mechanical Properties					
**Study**	σ(GPa)	ε	µ ^a^ (GPa)	υ ^b^	B ^c^ (GPa)
**Expt. by (Bartels et al.)**	−0.1757	−0.00523	14.91	0.24	24.71
**Expt. by (Barsch et al.)**	−0.1754	−0.00523	14.90	0.24	24.73
**Expt. by (Charles et al.)**	−0.1949	−0.00523	16.10	0.23	25.13
**Theory. by (Anderson et al.)**	−0.1771	−0.00523	14.93	0.25	25.30
**This study**	−0.1867	−0.00523	15.60	0.23	24.54

H = 2C_44_ + C_12_ − C_11_ [65]; (a) Shear modulus: $µ=C_{44}-\frac{1}{5}H$ [66]; (b) Poisson’s ratio: υ = $\frac{C_{12-\;\frac{H}{5}}}{2\left(C_{12}+C_{44}-2\;\frac{H}{5}\right)}$ [65]; (c) Bulk modulus: B = $\frac{C_{11}+2C_{12}}{3}$ [67].

## Supplementary Materials

The following are available online at https://www.mdpi.com/1996-1944/13/19/4380/s1, Figure S1: Geometry and the situation of involved atoms in diffracted planes (d) (311), (e) (222), (f) (400), (g) (331), (h) (420), (i) (422) and (j) (511) related to the NaCl.
