# Supplementary material for: Measurement Modulus of Elasticity Related to the Atomic Density of Planes in Unit Cell of Crystal Lattices
Source: Materials (Basel). 2020 Oct 1;13(19):4380. doi: 10.3390/ma13194380 (PMC7579002; doi:10.3390/ma13194380)
Supplement: Supplementary file 1 [file materials-13-04380-s001.pdf]

Supporting Information

d)

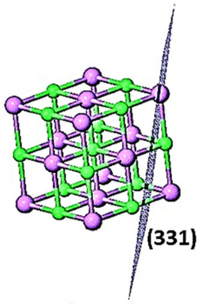

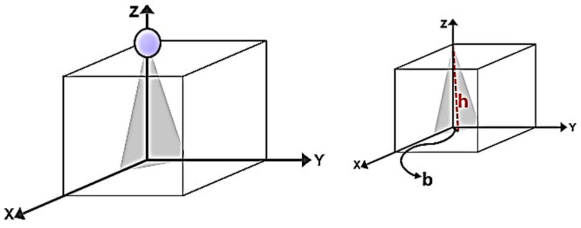

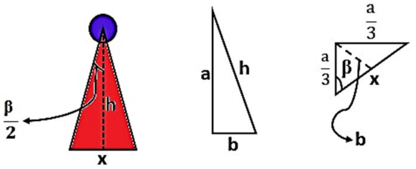

$(x)^2 = (\frac{a}{3})^2 + (\frac{a}{3})^2 = (\frac{5.64}{3})^2 + (\frac{5.64}{3})^2 \longrightarrow x = 2.65$

$\sin\beta = \frac{\frac{a}{3}}{x} = \frac{1.88}{2.65} = 0.70, \quad \beta = 44.42$

$\sin\beta = \frac{b}{\frac{a}{3}} = \frac{b}{1.88} = 0.70, \quad b = 1.31$

$(h)^2 = (a)^2 + (b)^2 = (5.64)^2 + (1.31)^2 \longrightarrow h = 5.78$

area of the plane (331): $S = \frac{\text{height} \times \text{width}}{2} = \frac{(h) \times (x)}{2} = \frac{(5.78) \times (2.65)}{2} = 7.65$

$\frac{\beta}{360} = \frac{24.80}{360} = 0.06$

$\tan\frac{\beta}{2} = \frac{\frac{x}{2}}{h} = \frac{\frac{2.65}{2}}{5.78} = 0.22 \longrightarrow \frac{\beta}{2} = 12.40, \ \beta = 24.80$

number of atoms in the plane (331) × area of each atom in the plane (331) $= [(1 \times 0.03)] \times \pi(r_{Cl^-})^2 = 0.06 \times \pi(1.81)^2 = 0.61$

planer density $= \frac{\text{number of atoms in the plane (331)} \times \text{area of each atom in the plane (331)}}{\text{area of the plane (331)}} = \frac{0.61}{7.65} = 0.07$

e)

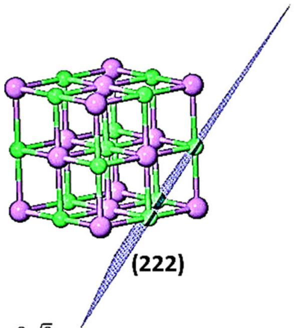

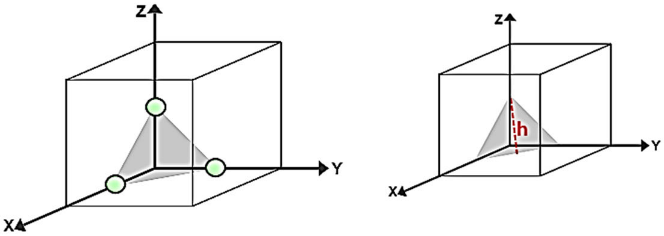

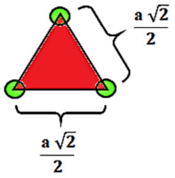

$(\frac{a\sqrt{2}}{2})^2 = (h)^2 + (\frac{a\sqrt{2}}{4})^2 \longrightarrow (3.97)^2 = (h)^2 + (1.98)^2 \longrightarrow h = 3.44$

area of the plane (222): $S = \frac{\text{height} \times \text{width}}{2} = \frac{(h) \times \frac{a\sqrt{2}}{2}}{2} = \frac{3.44 \times 3.97}{2} = 6.82$

number of atoms in the plane (222) × area of each atom in the plane (222) $= \left[\left(3 \times \frac{1}{6}\right)\right] \times \pi(r_{Na^+})^2 = \left[\left(3 \times \frac{1}{6}\right) \times \pi\,(0.97)^2\right] = 1.47$

planer density $= \frac{\text{number of atoms in the plane (222)} \times \text{area of each atom in the plane (222)}}{\text{area of the plane (222)}} = \frac{1.47}{6.82} = 0.21$

f)

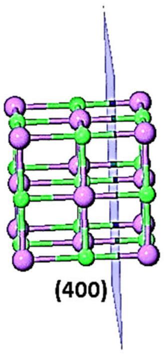

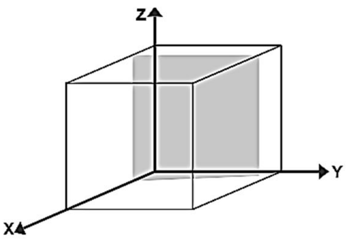

There is not atom in the plane (400)

g)

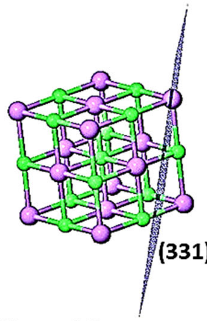

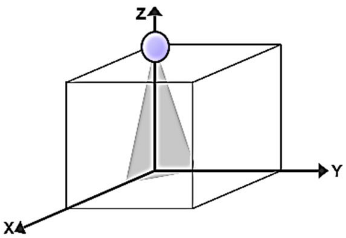

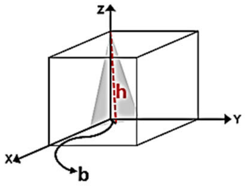

$(x)^2 = (\frac{a}{3})^2 + (\frac{a}{3})^2 = (\frac{5.64}{3})^2 + (\frac{5.64}{3})^2 \longrightarrow x = 2.65$

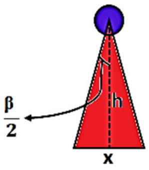

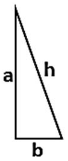

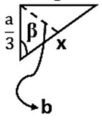

$\sin\beta = \frac{\frac{a}{3}}{x} = \frac{1.88}{2.65} = 0.70, \quad \beta = 44.42$

$\sin\beta = \frac{b}{\frac{a}{3}} = \frac{b}{1.88} = 0.70, \quad b = 1.31$

$(h)^2 = (a)^2 + (b)^2 = (5.64)^2 + (1.31)^2 \longrightarrow h = 5.78$

area of the plane (331): $S = \frac{\text{height} \times \text{width}}{2} = \frac{(h) \times (x)}{2} = \frac{(5.78) \times (2.65)}{2} = 7.65$

$\frac{\beta}{360} = \frac{12.40}{360} = 0.03$

$\tan\frac{\beta}{2} = \frac{\frac{x}{2}}{h} = \frac{\frac{2.65}{2}}{5.78} = 0.22 \longrightarrow \beta = 12.40$

number of atoms in the plane (331) × area of each atom in the plane (331) $= [(1 \times 0.03)] \times \pi(r_{Cl^-})^2 = 0.03 \times \pi(1.81)^2 = 0.30$

planer density $= \frac{\text{number of atoms in the plane (331)} \times \text{area of each atom in the plane (331)}}{\text{area of the plane (331)}} = \frac{0.30}{7.65} = 0.03$

h)

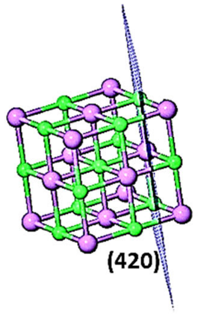

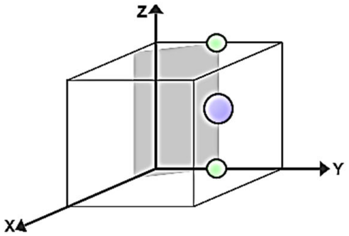

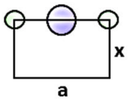

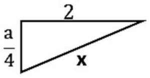

$(x)^2 = (\frac{a}{4})^2 + (\frac{a}{2})^2 = (\frac{5.64}{4})^2 + (\frac{5.64}{2})^2 \longrightarrow x = 3.15$

area of the plane (420): $S = a \times x = 5.64 \times 3.15 = 17.76$

number of atoms in the plane (420) × area of each atom in the plane (420) $= \left[\left(2 \times \frac{1}{4}\right)\right] \times \pi(r_{Na^+})^2 + \left[\left(1 \times \frac{1}{2}\right)\right] \times \pi(r_{Cl^-})^2$

$= \frac{1}{2} \times \pi(0.97)^2 + \frac{1}{2} \times \pi(1.81)^2 = 1.47 + 5.14 = 6.61$

planer density $= \frac{\text{number of atoms in the plane (420)} \times \text{area of each atom in the plane (420)}}{\text{area of the plane (420)}} = \frac{6.61}{17.76} = 0.37$

i)

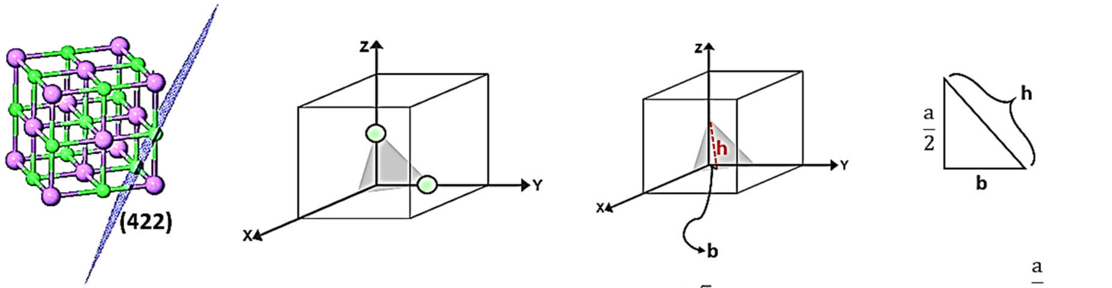

$x^2 = (\frac{a}{2})^2 + (\frac{a}{4})^2 \longrightarrow x = 3.15$

$\sin\alpha = \frac{\frac{a}{2}}{x} = \frac{2.82}{3.15} = 0.89 \longrightarrow \alpha = 62.87$

$\sin\alpha = \frac{b}{(\frac{a}{4})} = \frac{b}{1.41} = 0.89 \longrightarrow b = 1.25$

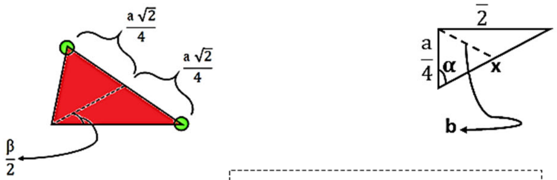

$h^2 = (\frac{a}{2})^2 + (b)^2 = (\frac{5.64}{2})^2 + (1.25)^2 \longrightarrow h = 3.08$

$\frac{180 - \beta}{2} = \frac{180 - 78.10}{2} = 50.95$

area of the plane (422): $S = \frac{\text{height} \times \text{width}}{2} = \frac{(x) \times (h)}{2} = \frac{(3.15) \times (3.08)}{2} = 4.85$

$\frac{50.95}{360} = 0.14$

$\sin\frac{\beta}{2} = \frac{\frac{a\sqrt{2}}{4}}{x} = \frac{1.98}{3.15} = 0.63, \quad \frac{\beta}{2} = 39.05, \quad \beta = 78.10$

number of atoms in the plane (422) × area of each atom in the plane (422) $= [(2 \times 0.14)] \times \pi(r_{Na^+})^2 = [(2 \times 0.14)] \times \pi(0.97)^2 = 0.82$

planer density $= \frac{\text{number of atoms in the plane (422)} \times \text{area of each atom in the plane (422)}}{\text{area of the plane (422)}} = \frac{0.82}{4.85} = 0.16$

j)

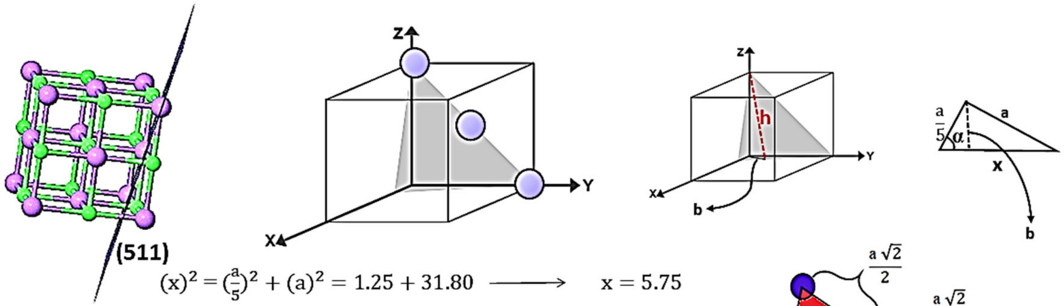

$(x)^2 = (\frac{a}{5})^2 + (a)^2 = 1.25 + 31.80 \longrightarrow x = 5.75$

$\sin\alpha = \frac{a}{x} = \frac{5.64}{5.75} = 0.98 \longrightarrow \alpha = 78.52$

$\sin\alpha = \frac{b}{\frac{a}{5}} = \frac{b}{1.12} = 0.98 \longrightarrow b = 1.09$

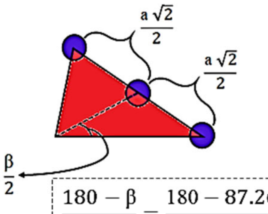

$(h)^2 = (a)^2 + (b)^2 = (5.64)^2 + (1.09)^2 \longrightarrow h = 5.74$

$\frac{180 - \beta}{2} = \frac{180 - 87.26}{2} = 46.37$

area of the plane (422): $S = \frac{\text{height} \times \text{width}}{2} = \frac{(h) \times (x)}{2} = \frac{(5.74) \times (5.75)}{2} = 16.50$

$\frac{\beta}{360} = \frac{46.37}{360} = 0.12$

$\sin\frac{\beta}{2} = \frac{\frac{a\sqrt{2}}{2}}{x} = \frac{3.97}{5.74} = 0.69, \quad \frac{\beta}{2} = 43.63, \quad \beta = 87.26$

number of atoms in the plane (511) × area of each atom in the plane (511) $= [(2 \times 0.12)] \times \pi(r_{Cl^-})^2 + \left[\left(1 \times \frac{1}{2}\right)\right] \times \pi(r_{Cl^-})^2 = [(2 \times 0.12)] \times \pi(1.81)^2 + \left[\left(1 \times \frac{1}{2}\right)\right] \times \pi(1.81)^2 = 7.60$

planer density $= \frac{\text{number of atoms in the plane (511)} \times \text{area of each atom in the plane (511)}}{\text{area of the plane (511)}} = \frac{7.60}{16.50} = 0.46$

Figure S1. Geometry and the situation of involved atoms in diffracted planes (**d**) (311), (**e**) (222), (**f**) (400), (**g**) (331), (**h**) (420), (**i**) (422) and (**j**) (511) related to the NaCl.
